# Physiotherapy Interventions in Lung Cancer Patients: A Systematic Review

**DOI:** 10.3390/cancers16050924

**Published:** 2024-02-25

**Authors:** Manuel Valdivia-Martínez, Miguel Ángel Fernández-Gualda, Elena Gallegos-García, Paula Postigo-Martin, María Fernández-González, Lucía Ortiz-Comino

**Affiliations:** 1 Faculty of Health Sciences, University of Granada, 18071 Granada, Spain; manuelvaldivia@correo.ugr.es (M.V.-M.); elenagallegos95@correo.ugr.es (E.G.-G.); mariafg@correo.ugr.es (M.F.-G.); 2 A02-Cuídate, Instituto de Investigación Biosanitaria ibs. GRANADA, 18012 Granada, Spain; paulapostigo@ugr.es (P.P.-M.); luciaoc@ugr.es (L.O.-C.); 3 Sports and Health Research Center (IMUDs), Parque Tecnológico de la Salud, 18007 Granada, Spain; 4 BIO277 Group, Department of Physical Therapy, Faculty of Health Sciences, University of Granada, 18071 Granada, Spain; 5 Faculty of Health Sciences Faculty (Melilla), University of Granada, 52005 Melilla, Spain

**Keywords:** lung cancer, lung capacity, functional capacity, physical exercise, respiratory physiotherapy, quality of life

## Abstract

**Simple Summary:**

Lung cancer represents 11.6% of the new cases and 18.4% of the total number of deaths caused by malignant tumors, and it is expected that in the next years the number of cases will continue to increase. Despite the improvements that can be obtained with medical treatment, several sequelae or side effects appear due to the treatment, reducing patients’ quality of life. Thus, this systematic review aims to study the effects of physiotherapy interventions to improve quality of life in patients with lung cancer.

**Abstract:**

Background: Lung cancer is a very common disease and leads to a series of sequelae such as reduced lung capacity or reduced functional capacity in patients, which are associated not only with the disease itself, but also with medical treatment. Thus, physiotherapeutic interventions are needed to improve quality of life and reduce these symptoms. Objectives: To find out the effects of physiotherapy on functional capacity, lung capacity, dyspnea, pain, and quality of life in lung cancer patients. Methods: A systematic review was carried out in five databases. Randomized clinical trials published between 2019–2023 were selected, in which the physiotherapeutic treatment was physical exercise and/or respiratory physiotherapy. Results: Nine articles were included, in which the total sample consisted of 635 lung cancer patients. When combined, respiratory physiotherapy and physical exercise improved functional capacity and lung capacity (*p* < 0.05). Dyspnea also improved, but less significance was shown in the included studies. Conclusions: Multimodal physiotherapy interventions may offer benefits for some lung cancer patients, but the extent and nature of these benefits may vary depending on the intervention applied. Therefore, it would be of great interest to carry out further scientific research to support this conclusion.

## 1. Introduction

Nowadays, lung cancer represents 11.6% of new cases and 18.4% of the total number of deaths caused by malignant tumors [1,2], and it is expected that in the next years the number of cases will continue to increase, due to both external (i.e., tobacco, electronic cigarettes, cigars, pipes, environmental smoke, ambient air pollution) and internal (i.e., genetic predisposition, chronic lung diseases, aged men, sedentarism, among others) risk factors [1]. Despite these data, the survival of patients with lung cancer is also increasing every year. A 5-year overall survival rate for all stages and types of lung cancer of 19.4% was reached in 2019 [2] and it is expected that due to scientific advances and early diagnosis, this figure will rise positively every year [1].

Scientific advances in oncological treatment improve this survival rate. Depending on the TNM stage [3], the invasion of adjacent structures, and the patient’s characteristics, surgery, chemotherapy, and/or radiotherapy may be included as treatment. Early TNM stages of small-cell lung cancer (SCLC) are considered operable, in which adjuvant treatment is administered [4,5]. However, in cases of early SCLC where pneumonectomy is recommended, specialists may exclude this treatment, as it is particularly unlikely to be in the patient’s favor. Moreover, lymph node sampling or lymph node dissection are mandatory in NSCLC treatment when the tumor is >2 cm [4]. Radiotherapy may be also prescribed, regardless of the tumor stage [6]; specifically, the combination of radiotherapy and chemotherapy is applied in patients with III NSCLC cancer stage [6]. However, when patients are not susceptible to surgical treatment, concurrent chemotherapy with radiotherapy is the treatment of choice, followed by immunotherapy. Finally, the standard treatment in metastatic NSCLC is immunotherapy with or without chemotherapy, mainly in tumors not harboring targetable mutations [5,7]. 

However, despite the improvements that can be obtained with medical treatment, several sequelae or side effects appear due to the treatment. Among these sequelae, the most common are decreased functional capacity and lung capacity, pain, dyspnea, and other non-related physical sequelae such as psychological distress. All these sequelae result indeed in a decrease in the patient’s quality of life (QoL) [8].

Nowadays, physiotherapy interventions such as physical exercise and respiratory physiotherapy are performed not only after the oncological treatment, but also as a preparation for the surgery [9], or even during adjuvant chemoradiotherapy [10]. These interventions have been shown to reduce the negative impact produced by oncological treatment, in both living with and beyond cancer [11]. Moreover, physiotherapy is included in many oncology guidelines [12,13], but its concordance is low. In the last few years, systematic reviews have shown positive effects of prehabilitation and rehabilitation in lung cancer [14,15,16], all concluding on the importance of future studies with more concordant interventions and results. 

To our knowledge, there are no updated systematic reviews unifying the effects of physical exercise and respiratory physiotherapy, in patients treated for lung cancer, during their hospital stay. Considering the significant social impact, the limited available information on the advantages of physical exercise for patients with this condition, and the scarcity of research on addressing the aftereffects of medical treatment, conducting a systematic review was considered highly important. The primary aim of this systematic review was to compile existing scientific evidence, shedding light on the importance of the treatment of the sequelae to achieve a good QoL for individuals dealing with lung cancer, to the greatest extent possible.

The main objective of this systematic review was to make known the effect of physical exercise and respiratory physiotherapy on functional capacity, lung capacity, and QoL in patients with lung cancer in each of the phases of the disease. The specific objectives were as follows:

(1) To highlight the effects of physiotherapy in improving dyspnea, pain, and fatigue in lung cancer patients.
(2) To describe which interventions are performed the most in lung cancer patients.

## 2. Materials and Methods

### 2.1. Study Design

This systematic review was conducted according to the Priority Reporting Items for Systematic Review and Meta-Analysis (PRISMA) [17] and registered by PROSPERO (CRD42024505504). The PICO strategy was used to describe the research question and thus search for randomized clinical trials (RCTs): (a) population: patients over 18 years of age diagnosed with lung cancer at any stage; (b) intervention: any physiotherapy intervention (e.g., physical exercise, respiratory physiotherapy); (c) comparison: traditional treatment by specialists in charge of the oncologic population; and (d) outcome: any outcome that could be improved with a physiotherapy intervention (e.g., functional capacity, lung capacity, quality of life).

### 2.2. Information Sources

To obtain the necessary information for this systematic review, a search was conducted between May 2023 and early July 2023. Different databases were consulted in this search, including Scopus, MEDLINE (via Pubmed), Web of Science (WOS), the Cochrane Library, and PEDro. These five academic and professional databases were chosen in consultation with a medical librarian. On the other hand, the references of different articles were also reviewed to find articles of interest. The last search to update our systematic review was performed in December 2023. 

### 2.3. Search Strategy

The research was carried out with the use of keywords, which were obtained through the web of health science descriptors (MeSH). In the case of this systematic review, these words were “lung cancer”, “carcinoma non-small-cell lung”, “carcinoma small-cell lung”, and “physiotherapy”. The combination of these terms with the Boolean operators (AND and OR) gave rise to the search equation used in this systematic review: [(lung cancer) OR (carcinoma small-cell lung) OR (carcinoma non-small-cell lung)], [(lung cancer) OR (carcinoma non-small-cell lung)] AND [(physiotherapy)]. This equation was used in the different databases already described, adapted to the specific characteristics of each of these databases. The different equations can be found in Table 1.

### 2.4. Inclusion Criteria

To select the articles, a series of criteria were considered. The articles included had to (a) be randomized controlled clinical trials (RCTs); (b) have free access through the Library of the University of Granada; (c) be published between 2019 and 2023; (d) have a sample of patients with lung cancer at any stage; and (e) be treatment-focused using respiratory physiotherapy and physical exercise.

### 2.5. Exclusion Criteria

Articles were excluded if (a) they were systematic reviews, communications to congresses, or any type of publication other than RCTs; (b) the control group received a physiotherapeutic intervention; (c) there was more than one group in the experimental intervention; and (d) a comparison between groups was not carried out. 

### 2.6. Selection Process

The software Rayyan (http://rayyan.qcri.org/, 8 February 2024) [18] was used to carry out the elimination of duplicates, the screening, and the selection of the studies to be included in this systematic review. The first step was to read the titles and abstracts of the articles, in order to discard all those that did not meet the inclusion criteria; then, a complete reading was performed of the remaining articles which were selected for this systematic review. Two reviewers (M.V.M. and M.A.F.G.) performed these steps individually, and, in case of doubt, a third reviewer (L.O.C.) was asked to make a decision. 

### 2.7. Data Extraction

Following the PICO strategy, the demographic and clinical data of all participants (age, sex, type of cancer, and oncological treatment) and the interventions of both the experimental and control groups (type, duration, frequency) were collected. Moreover, variables, instruments, and their results were registered. Finally, regarding the characteristics of the studies, the year of publication, type of study, and country in which the study was carried out were also collected.

### 2.8. Risk of Bias

The PEDro scale was used to assess the methodology of the studies included in this systematic review [19]. Depending on the obtained score, an excellent (9–10 points), good (6 to 8), normal (4 to 5), or low (less than 4) methodological quality was obtained [19].

## 3. Results

### 3.1. Descriptive Study

**Figure 1:**
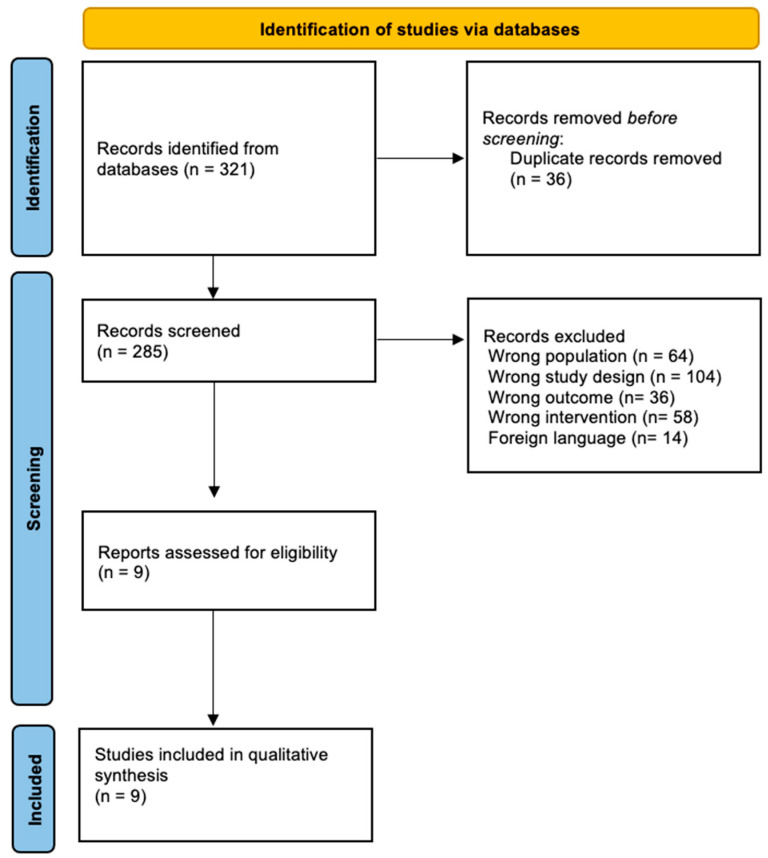
Flow diagram of the included studies.

After performing the methodological search in the different databases, a total of 321 articles were obtained, of which 36 were eliminated because they were duplicates. The next step was to apply the inclusion criteria, which resulted in a total of 276 articles being discarded, leaving 9 articles for complete analysis and thus for inclusion in the systematic review. Figure 1 shows the process of identification and study selection. The nine articles included in this review were all RCTs published in Sweden [20,21], China [22,23], Australia [24], France [25], Poland [26], Spain [27], and Taiwan [28]. 

### 3.2. Participants’ Characteristics

In this review, the total sample reached 635 lung cancer patients, with the most common type of lung cancer being NSCLC. The article with the largest sample was that of Jonnson et al. [20], with 107 patients, while the article with the smallest sample size was that of Gravier et al. [25], with only 36 patients. 

With respect to sex, the male sex was predominant. The age of the participants ranged from 56.2 ± 8.7 [22] years to 69 ± 8 years [21]. More information regarding participants’ characteristics can be found in Table 2.

### 3.3. Interventions

#### 3.3.1. Experimental Group

In general, the most frequent treatment was the combination of physical exercise and respiratory physiotherapy, in addition to the usual medical treatment. 

The maximum duration of physiotherapy was 8 weeks in the article by Edbrooke et al. [24] and the minimum was 3 days postoperatively in the article by M. Jonsson et al. [21]. 

Regarding the frequency of the rehabilitation program, the article with the highest frequency was that of Liu et al. [28] who performed the respiratory physiotherapy twice a day for six weeks and physiotherapy every day for six weeks. The lowest frequency was found in the article by Edbrooke et al. [24] who performed 2–3 sessions per week. In contrast, frequency was not mentioned in the article by Jonsson et al. [20]. Table 3 shows the characteristics of the experimental groups. 

#### 3.3.2. Control Group

In the studies included, control groups were only treated with the usual medical treatment for lung cancer patients, i.e., treatment by chemotherapy, radiotherapy, and/or surgery. Particularly, in the article by Fernández-Blanco et al. [27], the control group received only one medical consultation prior to surgery. Table 3 explains the information on the control group.

### 3.4. Studied Outcomes 

The most analyzed outcomes in the included studies were functional capacity [20,21,22,23,24,25,26,28] and lung capacity [20,21,22,25,26,28], followed by dyspnea [20,21,23,26]. Functional capacity was mainly [20,21,22,23,24,26,28] evaluated with the validated 6 minutes walking test (6MWT), which is commonly used not only in cancer patients [29] but also in patients with respiratory diseases to assess functional capacity [30]. For the assessment of the lung capacity, mainly spirometry [31] was used [20,21,22,25,26], as it allows the screening of the pulmonary function and the lung capacity itself [32]. Finally, dyspnea was evaluated in three articles [20,21,23] with the validated Modified Medical Research Council Dyspnea Scale mMRC [33]. 

Other secondary outcomes such as pain, QoL, anxiety and depression, functional disability, and prevalence of symptoms were also assessed with validated tools, as can be seen in Table 3. 

### 3.5. Effects of the Interventions

Results of the effects of the intervention on each variable analyzed in this study can be seen in Table 3. Specifically, the most analyzed variables in these studies were functional capacity [20,21,22,23,24,25,26,28] and lung capacity [20,21,22,25,26,28], followed by dyspnea [20,21,23,26]. Regarding the functional capacity, after the physiotherapeutic intervention, it increased with a significant difference intergroup (*p* < 0.05) in the studies of Liu, Liu Z, and Rutkowska et al. [22,26,28]. However, increments in the studies of Edbrooke et al., Jonssonª et al., and Jonsson et al. [20,21,24] were found, but with no significant differences between the two groups (*p* > 0.05). 

Lung capacity was evaluated in six articles, among which significant changes intergroup were obtained in three studies [22,26,28], as all pulmonary levels improved after completing the physiotherapy program. In contrast, lung capacity did not show a significant improvement in the studies of Gravier et al., Jonssonª et al., and Jonsson et al. [20,21,25], as lung volumes improved, but not enough to be statistically significant.

Dyspnea was studied in four studies, where significant improvements were found in the studies by Rutkowska et al. and Lu et al. [23,26]. In contrast, no significant intragroup improvements were found in the articles by Jonssonª et al. and Jonsson et al. [20,21]. 

Finally, other secondary outcomes were also assessed in the included studies. Pain in the experimental group only improved in one [27] of the three studies that evaluated this outcome. QoL was evaluated in two studies, one of them showing intragroup significant difference in favor of the experimental groups [24]. 

### 3.6. Risk of Bias

The PEDro scale was used to assess the methodological quality of the RCTs [19]. Among the articles included, the one with the highest methodological quality was that of Rutkowska et al. [26] with a maximum score of 10/10, which is considered to show an article of excellent methodological quality. In contrast, the articles with the lowest quality were those of Fernández-Blanco [27] and Jonsson^a^ et al. [21], both with a score of 6/10, which is considered to show a normal quality study. The results of the PEDro scale for the RCTs are shown in Table 4.

## 4. Discussion

This systematic review was performed with the aim of knowing the effects of physiotherapy, specifically physical exercise, and respiratory physiotherapy, on different variables such as lung capacity, functional capacity, and QoL in patients with lung cancer. After analyzing the results provided by the articles included in this review, we can confirm that physiotherapy improves different outcomes in patients with lung cancer. 

Functional capacity improved in four of the eight studies that evaluate this outcome [22,23,24,26]. These authors included multimodal physiotherapy interventions, including both respiratory physiotherapy and physical exercise, except for Edbrooke et al. [24], who only included aerobic exercise. In contrast, Gravier et al. [25] also developed a multimodal intervention; however, they did not use the 6MWT to evaluate this outcome but rather cardiopulmonary exercise testing. However, Gravier et al.’s control groups also included a rehabilitation program [25], whereas Edbrooke et al., Liu et al., Liu Zijia et al., and Rutkowska et al.’s control groups were usual care [22,23,24,26]. As most lung cancer patients are sedentary [34], it may be possible that any intervention, regardless of its duration, may improve this outcome. Moreover, aerobic training included in rehabilitation programs has been shown to improve functional capacity in lung morbidities [35], and other cancer populations [36,37,38,39], so lung cancer patients may also perceive this improvement. 

Regarding lung capacity, of the six studies that evaluated this outcome, only three reported significant improvements intergroup [22,26,28]. Although Gravier et al. [25] also included inspiratory muscle training in their experimental group, the interventions performed by Liu et al., Liu Zijia et al., and Rutkowska et al. [22,26,28] included more specific respiratory physiotherapy, which could enhance the results on this outcome, as respiratory physiotherapy causes an increase in lung volumes and therefore an improvement in this capacity [40]. This concords with the results obtained by Messaggi-Sartor et al. [41] which also evidenced improvements in lung capacity after a multimodal intervention in lung cancer patients. However, as that was a pilot randomized clinical trial, future research is advised to reinforce these results. 

Because of this physiotherapeutic multimodal treatment, statistically significant changes were seen in two of the four included studies that assessed dyspnea [23,26]. Although the intervention developed by Lu et al. [23] only included the active cycle of breathing techniques, it may be more effective to deal with dyspnea symptoms, when compared with the deep breathing exercises performed in the studies of Jonsson et al. and Jonsson^a^ et al. [20,21]. Dyspnea is suffered in almost half of patients with a diagnosis of lung cancer [42], and this symptom may be associated to other symptoms such as psychological distress and fatigue. Thus, interventions focused on the management of this outcome are needed, to secondarily improve these dyspnea-associated symptoms. 

Only three studies evaluated pain in lung cancer patients, and one showed statistically significant improvements in the experimental group [27]. Although pain is the most common symptom in cancer patients [43], lung cancer patients must deal with many other symptoms, while physiotherapy interventions focus on other outcomes. Moreover, most of the population studied in this systematic review received in-hospital interventions or at-home interventions before surgery or during chemoradiotherapy administration. Indeed, at the in-hospital interventions, usual care may also include medical treatment for cancer [43,44], while physiotherapy interventions may focus on other outcomes.

Finally, although other subjective outcomes such as QoL and depression and anxiety were assessed, only one study showed statistically significant improvements for QoL [24] and anxiety and depression [23]. Even if QoL was not assessed in all included studies, the favorable evolution of other outcomes (i.e., functional capacity and lung capacity) could have led to a significant improvement in the QoL with lung cancer thanks to physiotherapy, but it would be of great interest to carry out new studies to reinforce this affirmation.

Within the two types of treatment that we analyzed in this review (i.e., respiratory physiotherapy and physical exercise), we can say that the most effective treatment would be the combination of both, since it was the most widely used treatment in these studies and significant improvements in the results were obtained. Moreover, as physical exercise and respiratory physiotherapy show improvements when applied alone, it is understandable that when unifying them, results may also be positive.

### Limitations and Strengths

Despite the extensive analysis of the articles included in this review, some limitations can be pointed out. First, the number of articles is low (*n* = 9) due to the insufficient evidence available, which makes it likely that the results may change if the number of articles is increased. Regarding the articles themselves, as present limitations we can highlight that the work protocol is different in each article and that not all the articles evaluate the same variables. However, the present review also has strengths. One of the main strengths of this review is the use of validated tools to analyze the methodological quality of the studies. Another strength is that there is homogeneity among the main studied outcomes and the assessment tools, all of them being validated. Finally, the last strength to highlight is the timeliness of the articles included in the systematic review, since all of them have been published in the last 5 years.

Once this review has been completed, and considering the high incidence of lung cancer and the needs of these patients, it would be advisable to consider a series of guidelines such as the following: (a) a patient with lung cancer can benefit from physiotherapeutic treatment during all stages of medical treatment, that is, before, during, and after; (b) a treatment should be used where physical exercise and respiratory exercise are combined to benefit lung cancer patients; and (c) the therapeutic objective with this type of patients should be to improve their symptoms and therefore their QoL.

## 5. Conclusions

Physiotherapy interventions including physical exercise and respiratory physiotherapy have the potential to improve outcomes such as functional capacity and lung capacity in lung cancer patients. These improvements may be more significant with combined therapies when compared to a specific intervention. Other outcomes such as dyspnea, pain, and QoL seem to benefit from these interventions, but further research is advisable to confirm the results of this systematic review, as these results may vary depending on the specific intervention, the patients’ characteristics, and measured outcomes.

## Figures and Tables

**Table 1:** Search strategies in the consulted databases.

Database	Search Strategy
SCOPUS	((“lung cancer” OR “carcinoma non small-cell lung” OR “carcinoma small-cell lung”) AND (“physiotherapy”))
PUBMED	(((“lung cancer”) OR (“carcinoma non small-cell lung”)) OR (“carcinoma small-cell lung”)) AND (physiotherapy)
WEB OF SCIENCE	lung cancer (All Fields) OR carcinoma small-cell lung (All Fields) OR carcinoma non small-cell lung (All Fields) AND physiotherapy (All Fields)
COCHRANE	(lung cancer):ti,ab,kw OR (carcinoma small-cell lung):ti,ab,kw OR (carcinoma non small-cell lung):ti,ab,kw AND (physiotherapy):ti,ab,kw
PEDRO	[(lung cancer) OR (carcinoma small-cell lung) OR (carcinoma non small-cell lung)] AND [(physiotherapy)]

**Table 2:** Participants’ characteristics.

Author (Year)	Sample Size	Gender (Age, Years ± SD)	Cancer Type (Stage)	Medical Treatment
Edbrooke et al. (2019) [24]	92	Male: 55.4% Female: 45.6% (CG: 62.5 ± 10.9 EG: 64.6 ± 13.4)	LC	CT and/or RT
Gravier et al. (2022) [25]	36	Male: 64% Female: 36% 65–68 ± 8	NSCLC	Neoadjuvant RCT
Fernández-Blanco et al. (2022) [27]	71	Male: 61.97% Female: 38.03% (CG: 61.1 ± 11.9; EG: 64.1 ± 15.4)	LC	Surgery
Jonsson et al. (2019) [21]	94	Male: 47.9% Female: 52.1% (CG: 68 ± 8; EG: 69 ± 8)	NSCLC	Surgery
Jonsson et al. (2019) [20]	107	Male: 43.9% Female: 56.1% (CG: 68.4 ± 8.3; EG: 68.7 ± 7.4)	LC	Surgery
Liu et al. (2020) [28]	54	Male: 40.74% Female: 59.26% (CG: 66.3 ± 7.9; EG: 64.2 ± 5.9)	LC (IA-IIIA stages)	Surgery
Liu Z et al. (2020) [22]	73	Male: 31.5% Female: 68.5% (CG: 56.2 ± 8.7; EG:56.2 ± 10.3)	LC	Surgery
Lu et al. (2022) [23]	68	Male: 41.18% Female: 58.82% (CG: 57.03 ± 12.34; EG: 62.12 ± 8.03)	LC	Surgery
Rutkowska et al. (2019) [26]	40	Male: 100% (CG: 59.1 ± 6.8; EG: 61.3 ± 8.8)	NSCLC (IIIB-IV stages)	CT

CG: control group; CT: chemotherapy; EG: experimental group; LC: lung cancer; NSCLC: non-small-cell lung cancer; RT: radiotherapy; SD: standard deviation.

**Table 3:** Characteristics of the included studies.

Author (Year)	Sample Size	Control Group	Experimental Group	Duration	Outcomes	Results
Edbrooke et al. (2019) [24]	92	Usual care	**Monitored home-based rehabilitation**: (1) Moderate intensity aerobic (rating “4” on a Borg Dyspnea Scale) (2) Resistance exercise (80% of the 10RM)	2–3 sessions a week during 8 weeks	**FC** (6MWT) **QoL** (HrQol) **Symptoms** (MDADI)	**Between groups** **FC:** EG = CG (*p* > 0.05) **QoL:** EG > CG (*p* = 0.001) **Symptoms:** EG > CG (*p* = 0.001) **Within groups** Not reported
Gravier et al. (2022) [25]	36	Long regimen prehabilitation sessions (3 90′ sessions per week for 5 weeks)	**Short regimen of prehabilitation multimodal sessions:** (1) Moderate aerobic endurance training (2) Peripheral muscle strengthening (60–70% of the 1RM) (3) Inspiratory muscle training (4) Support and education	5 90′ sessions per week for 3 weeks	**FC** (CPET) **LCap** (spirometry) **QoL** (EORTC-QLQ-C30/LC13)	No significant changes in any of the assessed outcomes (*p* > 0.05)
Fernández-Blanco et al. (2022) [27]	71	Usual care	**Preoperative respiratory physiotherapy:** (1) Directed breathing (2) Incentive spirometry (3) Expiratory flow increase (4) Wound protection in coughing (5) Combination of physical therapy and aerosol therapy (6) Mobilization of the upper limb	5 sessions per week for 4 weeks	**Pain** (VAS) **PAL**	**Between groups** Pain: EG > CG (*p* = 0.005) PAL: EG > CG (*p* < 0.05) **Within groups** Not reported
Jonsson, et al. (2019) [21]	94	Usual care	**In-hospital physiotherapy:** (1) Ambulation (2) Mobility (3) Breathing exercises	5 20–30′ sessions during their hospital stay	**FC** (6MWT, IPAQ-E) **LCap** (spirometry) **Dyspnea** (mMRC) **Pain** (NRS)	**Between groups** No significant changes in any of the assessed outcomes (*p* > 0.05) **Within groups** FC: IPAQ-E improved in EG (*p* = 0.047)
Jonsson et al. (2019) [20]	107	Usual care	**Pre- and postoperative in-hospital physiotherapy + standard care:** (1) Individually adapted early mobilization (2) Deep breathing exercises (3) Exercises for thoracic and shoulder range of motion	1–2 sessions of 10–30′ per day, 6 days a week, during their hospital stay	**FC** (6MWT) **LCap** (spirometry) **Dyspnea** (mMRC) **Pain** (VAS)	No significant changes in any of the assessed outcomes (*p* > 0.05)
Liu et al. (2020) [28]	54	Usual Care	**Multimodal intervention:** (1) Moderate aerobic training (50–70% of the patient’s exercise capacity) (2) Respiratory physiotherapy	Respiratory physiotherapy: twice a day for 6 weeksAerobic exercise: 80′ a day for 6 weeks	**FC** (6MWT) **LCap** (MEP, MIP, LEV)	**Between groups** **FC:** EG > CG (*p* < 0.01) **LCAP:** EG > CG (*p* < 0.05) **Within groups** Not reported
Liu Z et al. (2020) [22]	73	Usual Care	**Multimodal prehabilitation intervention:** (1) Moderate exercise training (aerobic and resistance exercise) (2) Respiratory physiotherapy (3) Nutrition counseling	Exercise training: 30′ 3 days a week for 2 weeksRespiratory physiotherapy: 10′ twice daily for 2 weeks	**FC** (6MWT) **LCap** (spirometry) **Functional disability** (WHODAS 2.0) **Anxiety and depression** (HADS) **Short-term recovery quality** (QoR-9)	**Between groups** **FC:** EG > CG (*p* < 0.001) **LCap (FVC):** EG > CG (*p* = 0.021) **Within groups** Not reported
Lu et al. (2022) [23]	68	Usual Care	**Respiratory Physiotherapy - ACBT training:** (1) Breathing control (2) Thoracic expansion exercises (3) Forced expiration technique	20′ twice a day, during their hospital stay, before and after surgery	**FC** (6MWT) **Dyspnea** (mMRC) **Anxiety and depression** (HADS)	**Between groups** **Dyspnea:** EG > CG (*p* < 0.05) **Anxiety and depression:** EG > CG (*p* < 0.05) **Within groups** Not reported
Rutkowska et al. (2019) [26]	40	Usual care	**Multimodal intervention:** (1) 30’ of fitness and respiratory exercises (2) Specific respiratory exercises (3) Moderate aerobic training (30–80% of the peak work rate) (4) Moderate resistance exercise (40–70% of the 1RM) (5) Nordic walking for 45’ (6) Relaxation training	5 90′ sessions per week for 6 weeks	**FC** (6MWT, Fullerton test) **LCap** (spirometry) **Dyspnea** (Borg scale)	**Between groups** **FC**: (6MWT, TUG); EG > CG (*p* = 0.01) **LCap**: EG > CG (*p* < 0.05) **Dyspnea**: EG > CG (*p* = 0.04) **Within groups** Not reported

1RM: 1 repetition maximum; 10RM: 10 repetition maximum; 6MWT: six minutes walking test; ACBT: active cycle of breathing techniques; CG: control group; COPDAT: Chronic Obstructive Pulmonary Disease Assessment Test; CPET: cardiopulmonary exercise testing; EG: experimental group; EORTC-QLQ: European Organization for Research and Treatment of Cancer; FC: functional capacity; FVC: forced vital capacity; GHQ-12: General Health Questionnaire; HrQoL: health-related quality of life; IPAQ-E: International Physical Activity Questionnaire Modified for the Elderly; LCap: lung capacity; LEV: lung expansion volume; MDADI: MD Anderson Symptom Inventory; MEP: maximum expiratory pressure; MIP: maximum inspiratory pressure; mMRC: Modified Medical Research Council Dyspnea Scale; NRS: numeric rating scale; PAL: postoperative air leak; POS: postoperative pulmonary rehabilitation; PPO: pre- and postoperative pulmonary rehabilitation; PRE: preoperative pulmonary rehabilitation; PSQI: Pittsburgh Sleep Quality Index; QoL: quality of life; ROM: range of motion; SBQ: shortness of breath questionnaire; SF-16: Short Form-16; SFT: Senior Fitness Test; TUG: Timed Up and Go; VAS: visual analogue scale.

**Table 4:** Individual risk of bias.

Author, Year	1	2	3	4	5	6	7	8	9	10	11	Total
Edbrooke et al. (2019) [24]	Y	Y	Y	Y	N	N	Y	Y	Y	Y	Y	8/10
Gravier et al. (2022) [25]	Y	Y	Y	Y	Y	N	N	Y	Y	Y	N	7/10
Fernández-Blanco et al. (2022) [27]	Y	Y	Y	Y	N	N	N	Y	Y	Y	N	6/10
Jonsson et al. (2019) [21]	Y	Y	Y	Y	Y	N	Y	N	N	Y	N	6/10
Jonsson et al. (2019) [20]	Y	Y	Y	Y	Y	N	Y	Y	Y	Y	N	8/10
Liu et al. (2020) [28]	Y	Y	Y	Y	N	N	Y	N	Y	Y	N	7/10
Liu Zijia et al. (2020) [22]	Y	Y	Y	Y	N	N	Y	Y	Y	Y	N	8/10
Lu et al. (2022) [23]	Y	Y	Y	Y	N	N	Y	Y	Y	Y	N	8/10
Rutkowska et al. (2019) [26]	Y	Y	Y	Y	Y	Y	Y	Y	Y	Y	Y	10/10

1. Eligibility criteria were specified; 2. subjects were randomly allocated to groups (in a crossover study, subjects were randomly allocated an order in which treatments were received); 3. allocation was concealed; 4. the groups were similar at baseline regarding the most important prognostic indicators; 5. there was blinding of all subjects; 6. there was blinding of all therapists who administered the therapy; 7. there was blinding of all assessors who measured at least one key outcome; 8. measures of at least one key outcome were obtained from more than 85% of the subjects initially allocated to groups; 9. all subjects for whom outcome measures were available received the treatment or control condition as allocated or, where this was not the case, data for at least one key outcome were analyzed by “intention to treat”; 10. the results of between-group statistical comparisons are reported for at least one key outcome; 11. the study provides both point measures and measures of variability for at least one key outcome.

## References

[1] Bade B.C., Dela Cruz C.S. (2020). Lung Cancer 2020: Epidemiology, Etiology, and Prevention. Clin. Chest Med..

[2] Sung H., Ferlay J., Siegel R.L., Laversanne M., Soerjomataram I., Jemal A., Bray F. (2021). Global Cancer Statistics 2020: GLOBOCAN Estimates of Incidence and Mortality Worldwide for 36 Cancers in 185 Countries. CA Cancer J. Clin..

[3] Chansky K., Detterbeck F.C., Nicholson A.G., Rusch V.W., Vallières E., Groome P., Kennedy C., Krasnik M., Peake M., Shemanski L. (2017). The IASLC Lung Cancer Staging Project: External Validation of the Revision of the TNM Stage Groupings in the Eighth Edition of the TNM Classification of Lung Cancer. J. Thorac. Oncol..

[4] Menéndez P., Sánchez-Torres J.M., Bartolomé A., Bravo J.L., Caballero-Guerra P., Calzas-Rodríguez J., Cortés-Funes H., Díaz-Hellín V., Doñado-Uña J.R., Enguita A.B. (2009). OncoSur Guía Clínica de Diagnóstico y Tratamiento Del Cáncer de Pulmón.

[5] Pirker R. (2020). Chemotherapy Remains a Cornerstone in the Treatment of Nonsmall Cell Lung Cancer. Curr. Opin. Oncol..

[6] Vinod S.K., Hau E. (2020). Radiotherapy Treatment for Lung Cancer: Current Status and Future Directions. Respirology.

[7] Hanna N.H., Robinson A.G., Temin S., Baker S., Brahmer J.R., Ellis P.M., Gaspar L.E., Haddad R.Y., Hesketh P.J., Jain D. (2021). Therapy for Stage IV Non-Small-Cell Lung Cancer with Driver Alterations: ASCO and OH (CCO) Joint Guideline Update. J. Clin. Oncol..

[8] Molassiotis A., Yates P., Yorke J. (2021). Editorial: Quality of Life and Side Effects Management in Lung Cancer Treatment. Front. Oncol..

[9] Cjl M., Rooijen V.S., Hjp F., Rmh R., Janssen L., Slooter G.D. (2022). Cochrane Library Cochrane Database of Systematic Reviews Prehabilitation versus No Prehabilitation to Improve Functional Capacity, Reduce Postoperative Complications and Improve Quality of Life in Colorectal Cancer Surgery. Emergencias.

[10] Medeiros Torres D., Koifman R.J., da Silva Santos S. (2022). Impact on Fatigue of Different Types of Physical Exercise during Adjuvant Chemotherapy and Radiotherapy in Breast Cancer: Systematic Review and Meta-Analysis. Support. Care Cancer.

[11] Smith S.R., Zheng J.Y., Silver J., Haig A.J., Cheville A. (2020). Cancer Rehabilitation as an Essential Component of Quality Care and Survivorship from an International Perspective. Disabil. Rehabil..

[12] Stout N.L., Santa Mina D., Lyons K.D., Robb K., Silver J.K. (2021). A Systematic Review of Rehabilitation and Exercise Recommendations in Oncology Guidelines. CA. Cancer J. Clin..

[13] Shallwani S.M., King J., Thomas R., Thevenot O., De Angelis G., Aburub A.S., Brosseau L. (2019). Methodological Quality of Clinical Practice Guidelines with Physical Activity Recommendations for People Diagnosed with Cancer: A Systematic Critical Appraisal Using the AGREE II Tool. PLoS ONE.

[14] Driessen E.J., Peeters M.E., Bongers B.C., Maas H.A., Bootsma G.P., Van Meeteren N.L., Janssen-Heijnen M.L. (2017). Effects of Prehabilitation and Rehabilitation Including a Home-Based Component on Physical Fitness, Adherence, Treatment Tolerance, and Recovery in Patients with Non-Small Cell Lung Cancer: A Systematic Review. Crit. Rev. Oncol. Hematol..

[15] Codima A., das Neves Silva W., de Souza Borges A.P., de Castro G. (2021). Exercise Prescription for Symptoms and Quality of Life Improvements in Lung Cancer Patients: A Systematic Review. Support. Care Cancer.

[16] Wang Y.Q., Liu X., Yin Y.Y., Ma R.C., Yang Z., Cao H.P., Xie J. (2019). Effects of Home-Based Exercise Training for Patients With Lung Cancer. Oncol. Nurs. Forum.

[17] Liberati A., Altman D.G., Tetzlaff J., Mulrow C., Gøtzsche P.C., Ioannidis J.P., Clarke M., Devereaux P.J., Kleijnen J., Moher D. (2009). The PRISMA Statement for Reporting Systematic Reviews and Meta-Analyses of Studies That Evaluate Health Care Interventions: Explanation and Elaboration. Ann. Intern. Med..

[18] Ouzzani M., Hammady H., Fedorowicz Z., Elmagarmid A. (2016). Rayyan—A Web and Mobile App for Systematic Reviews. Syst. Rev..

[19] Maher C.G., Sherrington C., Herbert R.D., Moseley A.M., Elkins M. (2003). Reliability of the PEDro Scale for Rating Quality of Randomized Controlled Trials. Phys. Ther..

[20] Jonsson M., Ahlsson A., Hurtig-Wennlöf A., Vidlund M., Cao Y., Westerdahl E. (2019). In-Hospital Physiotherapy and Physical Recovery 3 Months After Lung Cancer Surgery: A Randomized Controlled Trial. Integr. Cancer Ther..

[21] Jonsson M., Hurtig-Wennlöf A., Ahlsson A., Vidlund M., Cao Y., Westerdahl E. (2019). In-Hospital Physiotherapy Improves Physical Activity Level after Lung Cancer Surgery: A Randomized Controlled Trial. Physiother.

[22] Liu Z., Qiu T., Pei L., Zhang Y., Xu L., Cui Y., Liang N., Li S., Chen W., Huang Y. (2020). Two-Week Multimodal Prehabilitation Program Improves Perioperative Functional Capability in Patients Undergoing Thoracoscopic Lobectomy for Lung Cancer: A Randomized Controlled Trial. Anesth. Analg..

[23] Lu H.B., Liu X., Wang Y.Q., Cao H.P., Ma R.C., Yin Y.Y., Song C.Y., Yang T.T., Xie J. (2022). Active Cycle of Breathing Technique: A Respiratory Modality to Improve Perioperative Outcomes in Patients With Lung Cancer. Clin. J. Oncol. Nurs..

[24] Edbrooke L., Aranda S., Granger C.L., Mcdonald C.F., Krishnasamy M., Mileshkin L., Clark R.A., Gordon I., Irving L., Denehy L. (2019). Multidisciplinary Home-Based Rehabilitation in Inoperable Lung Cancer: A Randomised Controlled Trial. Thorax.

[25] Gravier F.E., Smondack P., Boujibar F., Prieur G., Medrinal C., Combret Y., Muir J.F., Baste J.M., Cuvelier A., Debeaumont D. (2022). Prehabilitation Sessions Can Be Provided More Frequently in a Shortened Regimen with Similar or Better Efficacy in People with Non-Small Cell Lung Cancer: A Randomised Trial. J. Physiother..

[26] Rutkowska A., Jastrzebski D., Rutkowski S., Zebrowska A., Stanula A., Szczegielniak J., Ziora D., Casaburi R. (2019). Exercise Training in Patients With Non-Small Cell Lung Cancer During In-Hospital Chemotherapy Treatment: A Randomized Controlled Trial. J. Cardiopulm. Rehabil. Prev..

[27] Fernández-Blanco R., Rincón-García D., Valero-Alcaide R., Atín-Arratibel M.A., De Miguel-Diez J., Corrochano-Cardona R., Torres-Castro R., Moro-Tejedor M.N. (2023). Preoperative Respiratory Therapy in Patients Undergoing Surgery for Lung Cancer: A Randomized Controlled Trial. Physiother. Res. Int..

[28] Liu J.F., Kuo N.Y., Fang T.P., Chen J.O., Lu H.I., Lin H.L. (2021). A Six-Week Inspiratory Muscle Training and Aerobic Exercise Improves Respiratory Muscle Strength and Exercise Capacity in Lung Cancer Patients after Video-Assisted Thoracoscopic Surgery: A Randomized Controlled Trial. Clin. Rehabil..

[29] Schmidt K., Vogt L., Thiel C., Jäger E., Banzer W. (2013). Validity of the Six-Minute Walk Test in Cancer Patients. Int. J. Sports Med..

[30] Holland A.E., Spruit M.A., Troosters T., Puhan M.A., Pepin V., Saey D., McCormack M.C., Carlin B.W., Sciurba F.C., Pitta F. (2014). An Official European Respiratory Society/American Thoracic Society Technical Standard: Field Walking Tests in Chronic Respiratory Disease. Eur. Respir. J..

[31] Zhao N., Wu F., Peng J., Zheng Y., Tian H., Yang H., Deng Z., Wang Z., Li H., Wen X. (2022). Preserved Ratio Impaired Spirometry Is Associated with Small Airway Dysfunction and Reduced Total Lung Capacity. Respir. Res..

[32] Rivero-Yeverino D. (2019). Spirometry: Basic Concepts. Rev. Alerg. Mex..

[33] Sunjaya A., Poulos L., Reddel H., Jenkins C. (2022). Qualitative Validation of the Modified Medical Research Council (MMRC) Dyspnoea Scale as a Patient-Reported Measure of Breathlessness Severity. Respir. Med..

[34] D’silva A., Gardiner P.A., Boyle T., Bebb D.G., Johnson S.T., Vallance J.K. (2018). Associations of Objectively Assessed Physical Activity and Sedentary Time with Health-Related Quality of Life among Lung Cancer Survivors: A Quantile Regression Approach. Lung Cancer.

[35] Dowman L., Hill C.J., May A., Holland A.E. (2021). Pulmonary Rehabilitation for Interstitial Lung Disease. Cochrane Database Syst. Rev..

[36] Reis A.D., Pereira P.T.V.T., Diniz R.R., de Castro Filha J.G.L., dos Santos A.M., Ramallo B.T., Filho F.A.A., Navarro F., Garcia J.B.S. (2018). Effect of Exercise on Pain and Functional Capacity in Breast Cancer Patients. Health Qual. Life Outcomes.

[37] Samuel S.R., Maiya A.G., Fernandes D.J., Guddattu V., Saxena P.P., Kurian J.R., Lin P.-J., Mustian K.M. (2019). Effectiveness of Exercise-Based Rehabilitation on Functional Capacity and Quality of Life in Head and Neck Cancer Patients Receiving Chemo-Radiotherapy. Support. Care Cancer.

[38] Sancho A., Carrera S., Arietaleanizbeascoa M., Arce V., Gallastegui M.M., March A.G., Sanz-Guinea A., Eskisabel A., Rodriguez L.L., Martín R.A. (2015). Supervised Physical Exercise to Improve the Quality of Life of Cancer Patients: The Eficancer Randomised Controlled Trial. BMC Cancer.

[39] Mendizabal-Gallastegui N., Arietaleanizbeaskoa M.S., Latorre P.M., García-Álvarez A., Sancho A., Iruarrizaga E., López-Vivanco G., Grandes G. (2023). Nurse-Supervised Exercise for People with Stage IV Cancer: The EFICANCER Randomized Clinical Trial. Semin. Oncol. Nurs..

[40] Rodrigues A., Castro G.M., Jácome C., Langer D., Parry S.M., Burtin C. (2020). Current Developments and Future Directions in Respiratory Physiotherapy. Eur. Respir. Rev..

[41] Messaggi-Sartor M., Marco E., Martínez-Téllez E., Rodriguez-Fuster A., Palomares C., Chiarella S., Muniesa J.M., Orozco-Levi M., Barreiro E., Güell M.R. (2019). Combined Aerobic Exercise and High-Intensity Respiratory Muscle Training in Patients Surgically Treated for Non-Small Cell Lung Cancer: A Pilot Randomized Clinical Trial. Eur. J. Phys. Rehabil. Med..

[42] Shin J.A., Kosiba J.D., Traeger L., Greer J.A., Temel J.S., Pirl W.F. (2014). Dyspnea and Panic Among Patients With Newly Diagnosed Non-Small Cell Lung Cancer. J. Pain Symptom Manage..

[43] Gül Ş.K., Tepetam H., Gül H.L. (2020). Duloxetine and Pregabalin in Neuropathic Pain of Lung Cancer Patients. Brain Behav..

[44] Matsuoka H., Iwase S., Miyaji T., Kawaguchi T., Ariyoshi K., Oyamada S., Satomi E., Ishiki H., Hasuo H., Sakuma H. (2019). Additive Duloxetine for Cancer-Related Neuropathic Pain Nonresponsive or Intolerant to Opioid-Pregabalin Therapy: A Randomized Controlled Trial (JORTC-PAL08). J. Pain Symptom Manag..

